# Home blood pressure data visualization for the management of hypertension: using human factors and design principles

**DOI:** 10.1186/s12911-021-01598-4

**Published:** 2021-08-05

**Authors:** Pete Wegier, Jeffery L. Belden, Shannon M. Canfield, Victoria A. Shaffer, Sonal J. Patil, Michael L. LeFevre, K. D. Valentine, Mihail Popescu, Linsey M. Steege, Akshay Jain, Richelle J. Koopman

**Affiliations:** 1 Humber River Hospital, Toronto, ON Canada; 2 Institute of Health Policy, Management and Evaluation, University of Toronto, Toronto, ON Canada; 3 Department of Family and Community Medicine, University of Toronto, Toronto, ON Canada; 4 Department of Family and Community Medicine, University of Missouri-Columbia, Columbia, MO USA; 5 Department of Psychological Sciences, University of Missouri-Columbia, Columbia, MO USA; 6 Health Decision Sciences Center, Massachusetts General Hospital, Boston, MA USA; 7 Harvard Medical School, Boston, MA USA; 8 Department of Health Management and Informatics, University of Missouri-Columbia, Columbia, MO USA; 9 School of Nursing, University of Wisconsin-Madison, Madison, WI USA; 10 Department of Electrical and Computer Engineering, University of Missouri-Columbia, Columbia, MO USA

**Keywords:** Data visualization, Interface design, Blood pressure, Hypertension, Shared decision making

## Abstract

**Background:**

Home blood pressure measurements have equal or even greater predictive value than clinic blood pressure measurements regarding cardiovascular outcomes. With advances in home blood pressure monitors, we face an imminent flood of home measurements, but current electronic health record systems lack the functionality to allow us to use this data to its fullest. We designed a data visualization display for blood pressure measurements to be used for shared decision making around hypertension.

**Methods:**

We used an iterative, rapid-prototyping, user-centred design approach to determine the most appropriate designs for this data display. We relied on visual cognition and human factors principles when designing our display. Feedback was provided by expert members of our multidisciplinary research team and through a series of end-user focus groups, comprised of either hypertensive patients or their healthcare providers required from eight academic, community-based practices in the Midwest of the United States.

**Results:**

A total of 40 participants were recruited to participate in patient (N = 16) and provider (N = 24) focus groups. We describe the conceptualization and development of data display for shared decision making around hypertension. We designed and received feedback from both patients and healthcare providers on a number of design elements that were reported to be helpful in understanding blood pressure measurements.

**Conclusions:**

We developed a data display for substantial amounts of blood pressure measurements that is both simple to understand for patients, but powerful enough to inform clinical decision making. The display used a line graph format for ease of understanding, a LOWESS function for smoothing data to reduce the weight users placed on outlier measurements, colored goal range bands to allow users to quickly determine if measurements were in range, a medication timeline to help link recorded blood pressure measurements with the medications a patient was taking. A data display such as this, specifically designed to encourage shared decision making between hypertensive patients and their healthcare providers, could help us overcome the clinical inertia that often results in a lack of treatment intensification, leading to better care for the 35 million Americans with uncontrolled hypertension.

## Background

Blood pressure (BP) control in the US is a public health problem. Hypertension is uncontrolled in about half of the 70 million US adults diagnosed with the disease [1–3] despite the existence of multiple drugs to help treat hypertension. The majority of BP measurements are currently gathered in physicians’ offices, as a part of regular visits, leading to a dearth of measurements. Variation is common, with BP rising and falling based on factors such as stress, physical activity, white-coat hypertension [4], and user error in clinical setting [5]. The uncertainty caused by this variation has been noted as a potential source of clinical inertia and confusion for patients facing hypertension treatment decisions [6–9].

Recent research has identified the important role of home BP measurements, considered equal—or even superior—to clinic BP measurements in their value in predicting cardiac risk [10, 11]. Home BP monitoring is becoming more common, with seamless uploading of home BP measurements directly into a patient’s electronic health record (EHR) on the horizon. With recent innovations in continuous personal heart rate monitoring (e.g., Apple Watch, Fitbit), a future of continuous BP monitoring may not be far behind. We believe this will transition both patients and healthcare providers from the current landscape of BP data scarcity to a future of data abundance and, if we are not prepared, data overload.

Currently, for patients who monitor their BP at home, the data they collect may not be utilized to its potential. Understanding tabular presentations of numerical data often requires significant cognitive resources, the format often hides context, and trends are more difficult to detect. Data visualizations are a highly effective way to communicate both quantitative and probabilistic information to patients, with a variety of formats being effective [12–14]. However, data visualizations need be neither novel nor complicated for patients to gain benefit—a simple bar graph on a home BP monitor has been shown to improve BP control, compared to a control group who used a BP monitor without the bar graph [15].

Despite the power of data visualization as a technique to communicate quantitative information, graphing capabilities in current EHR systems are limited [16]. Graphs and other visualizations are not the default mode of presentation, leaving users to discover the features on their own or not at all. Axes are often dynamically selected to fit the available data—such axes can end up displaying apparently great variation over a narrow range of values, when the axes should be anchored to meaningful baselines. Summary statistics are rare, leaving users to estimate values themselves. Finally, graphs tend to focus solely on the communication of raw data in isolation rather than presenting that data in the relevant clinical context (e.g., values presented without meaningful target or goal ranges).

Previously, we reported our efforts to determine the informational needs of patients and physicians to engage in shared decision making around hypertension using a single data display [17]. In that work, we reported the results of several focus groups with patients and healthcare providers on the informational needs of both groups, specifically what data would need to be present in a display so patients and providers could engage in shared decision making around hypertension management.

In this paper, we present the design and refinement of such a data display to be used by a patient and their healthcare provider simultaneously, during an office visit, to improve shared decision making around hypertension management. We aimed to address several problems currently plaguing patients and physicians about the display of blood pressure measurements: (1) patients lack clarity about goal BP values; (2) patients are concerned by outlier values despite the average BP being in the goal range and considered “controlled”; (3) patients’ overdependence on the most recent BP values; and (4) patients not understanding the temporal connection between medication changes and BP response, as BP and medications are typically not on the same screen, and onset of action delay is different for each medication. We used an iterative, rapid-prototyping, user-centred design methodology, involving both our core research team and the focus groups reported in the previous work [17]. We focused on visual cognition and human factors principles to drive the design of prototype displays, with many decisions being made based on feedback from patients and providers. We have tested many of the features we developed here in a series of experimental studies [18, 19]. Our goal for this paper is to share how these design decisions were made based on knowledge gathered from the focus groups and encourage further future development in this area.

## Methods

Our research and design team was assembled to develop an optimized BP data display to support clinical decision-making by patients and physicians. The team includes practicing family physicians; a physician member of Joint National Commission on Hypertension (JNC-8) and former chair of the United States Preventive Services Task Force (USPSTF) [20]; a data science engineer and medical informatician with skills in algorithmic processing and natural language generation; an industrial and systems engineer with expertise in human factors in healthcare systems; psychologists with considerable experience studying medical decision making; and mixed-methods researchers in health information technology use and changes in ambulatory practice. This team composition brought together multiple expert perspectives for guided discussions and allowed us to make informed, internally validated design decisions.

### Approach

Inherent in our approach was our mission to design for shared physician and patient use to inform shared decision making around hypertension. We conducted a total of five focus groups dedicated to reviewing prototype displays and iterating on their design, alternating between hypertensive patients, and family physicians and internal medicine physicians—focus groups 1, 3, and 5 were with patients, and focus groups 2 and 4 were with physicians. Analysis of focus group qualitative data took place in three phases: a team debrief, a rapid preliminary analysis immediately following each focus group, and final analysis of compiled data. Patient and physician participants returned for three confirmatory focus groups to view and comment on the final prototype.

The preliminary qualitative analysis following each focus group helped us identify participant responses to our designs and to allow for rapid iteration of our prototypes [21]. A more traditional and comprehensive thematic analysis [22] of the qualitative data was conducted by RJK and SMC, using a deductive realist approach. Additional details regarding the data collection and analysis, and the interview guide, can be found in a related paper [17]. Each of the quotes we selected to present here were chosen for two reasons: (1) the quotes represented common positions held by focus group participants; and (2) the quotes drove specific design decisions.

### Participants

The University of Missouri Health Sciences Institutional Review Board reviewed and approved this study and its human subjects’ participation and consent process. We recruited Family and Community Medicine (FCM) and General Internal Medicine (GIM) physicians from eight academic, community-based practices in the Midwest of the United States. We contacted their patients—aged 18 years or older, with a diagnosis of hypertension, and identified by their physicians as appropriate to participate in this study—by a mailed letter, signed by their physician. During each focus group, prototype displays were presented to all participants with minimal briefing to gauge the intuitiveness of each design. Participant demographics are presented in Table 1.

**Table 1:** Focus group participants' characteristics

Characteristics*	Patients	Physicians
*N*	16	24
*Gender—% (N)*		
Female	62 (10)	33 (8)
Male	38 (6)	67 (16)
*Age—M (SD)*	59 (17.6)	48 (13.6)
*Race—% (N)*		
White	88 (14)	92 (22)
Black/African American	6 (1)	4 (1)
Other	6 (1)	4 (1)
*Ethnicity—% (N)*		
Latino / Latina	0	0
*Education (patients—% (N))*		
Some college or greater	62 (10)	
High school or GED	19 (3)	
Less than high school	19 (3)	
*Years in practice (physicians—% (N))*		
Less than 5 years		29 (7)
6–20 years		33 (8)
21–30 years		21 (5)
More than 30 years		17 (4)

*Response options included additional categories. Only those reported by participants are included here

## Results

### Blood pressure plot

#### Style of plot

**Fig. 1:**
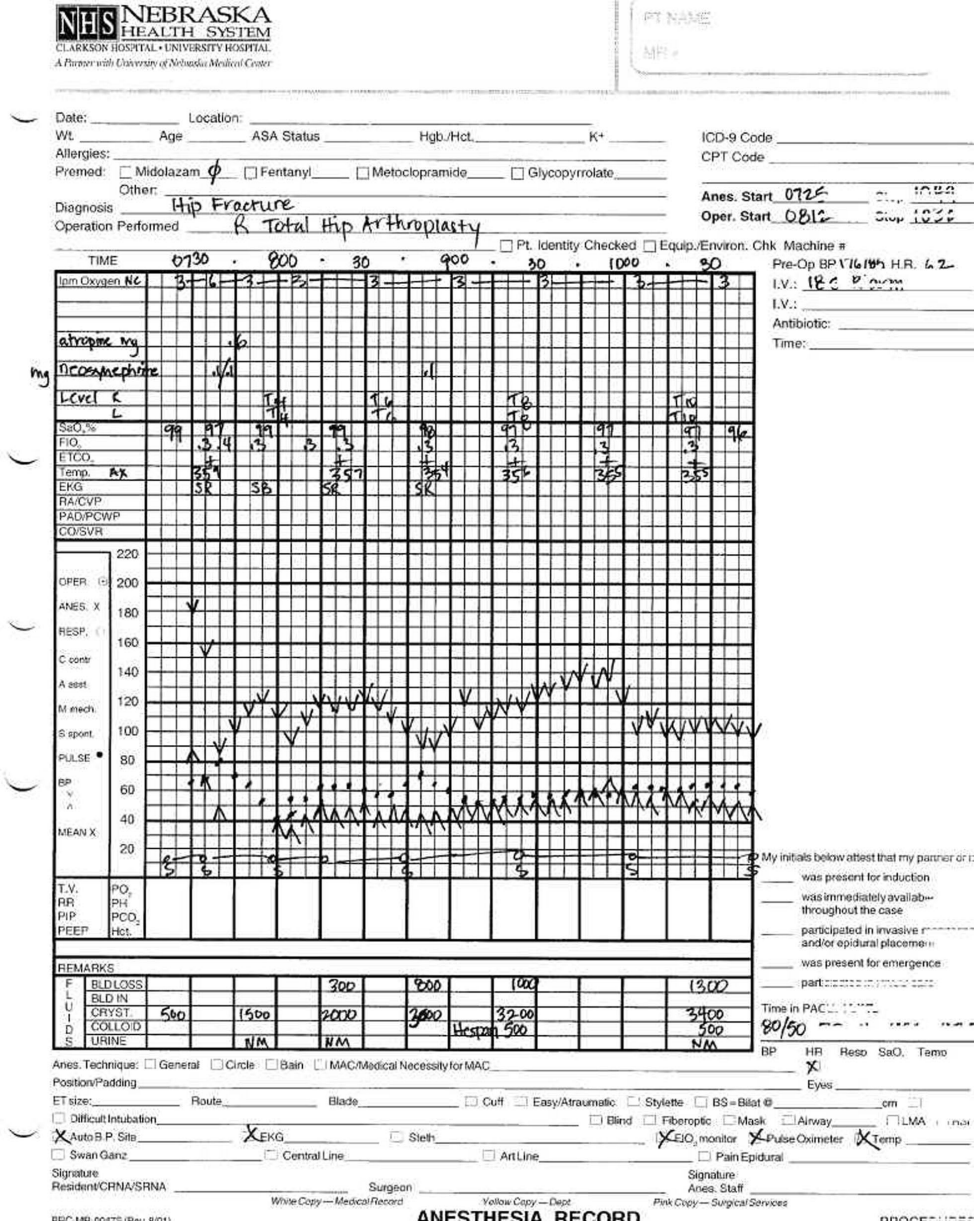
Sample anesthesia record showing the system of inverted carets to mark SBP and carets to mark DBP. Reproduced with permission from University of Nebraska Medical Center

Graphing blood pressure carries additional demands compared to a single-number measurement such as body weight. A single blood pressure measurement is a pair of numbers and considered as such by both clinicians and patients. We considered two potential models currently in use. First, anesthesiology professionals have graphed blood pressure with pen and paper using an inverted caret and caret to mark the systolic BP (SBP) and diastolic BP (DBP) values, respectively (Fig. 1). This allows for rapid annotation and high information density when BP values may be taken every few minutes. Second, electronic health records have sometimes adapted the anesthesiology visual display model to include a vertical connecting line to the DBP and SBP for ease of visual recognition. While common in anesthesia and critical care EHR displays, this style would be rarely seen and thus unfamiliar to users in the ambulatory setting. Rather than designing for novelty, we decided to stick with what would be most familiar to patients and used a simple line graph.

#### Denoting specific sources of BP readings

The raw data in the EHR should reveal the source of measurement (home, office, 24-h ambulatory, hospital, operative period, emergency medical services, etc.). We explored separate symbols to represent each of these sources. Additionally, we explored distinct symbols to differentiate confirmed/validated (thus more trustworthy) home BP measurements versus unconfirmed/unvalidated home measurements. “Home confirmed” refers to having clinical staff validate the accuracy of a patient’s cuff and measurement technique. Data trustworthiness depends on accurate and complete recordkeeping by the patient, the use of proper technique, and confirmation of that technique and results by office staff using the same equipment.

*“I have them come in with the cuff to see how well their cuff works with my cuff. I would think confirmed versus unconfirmed would be confirmed with the nurse runs through the machine, writes down some numbers from, you know, hard data, versus unconfirmed would be patients calling us in with some numbers, ‘cause they could make those up.”*

— **Round 4, GIM Physician 7**

However, we discovered in our focus groups that using a different visual symbol for each distinct data source added more complexity than needed for the task at hand: management of hypertension in the ambulatory setting giving priority to home BP values over office BP values. For example, inpatient or operative BP values were already recognizable by their markedly higher density—due to frequent measurement—and thus did not need symbols distinct from other non-home (clinical) BP measurements. Each new symbol gave the impression of importance; however, when everything is important, nothing stands out.

*“I’m not sure that differentiation matters to me that much.”*

**— Round 2, FCM Physician 19**

**Fig. 2:**
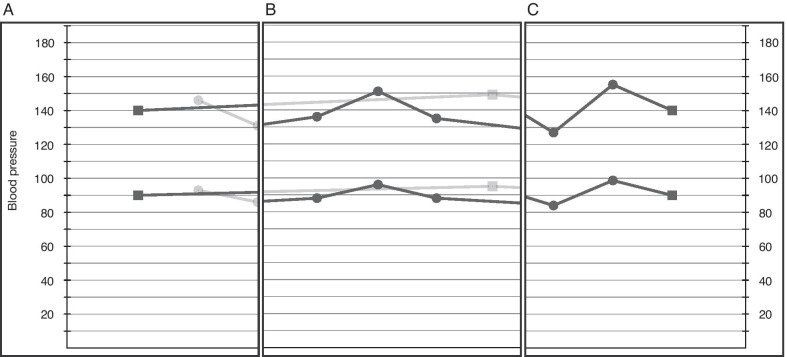
Examples of the different styles of BP plots we designed. **A** and **B** show the 4-line paradigm, in which office and home measurements are plotted on separate lines, with squares and circles denoting office and home measurements, respectively. Users could switch focus between the office and home lines—**A** shows the office measurements in focus and **B** shows the home measurements in focus. **C** shows our combined 2-line paradigm, with office and home values connected and differentiated by the shape of the data points (squares and circles for office and home measurements, respectively)

Currently, most BP records are primarily made up of office BP measurements, gathered during regular visits. We expect this to change in the future as internet-connected home BP cuffs become integrated with patient portals and EHRs. Additionally, it is likely that passive BP monitoring is in our future as the personal health device (e.g., Apple Watch, Fitbit) market is exploding in size [23]. We explored rendering office (Fig. 2A) and home (Fig. 2B) measurements as separate lines—a 4-line paradigm—within the same graph window, with controls to switch the focus between home and office BP measurements. Four lines had the potential for overlap and visual confusion, so our final design used a 2-line paradigm. An initial design connected only home BP measurements and left office BP measurements disconnected—to emphasize the importance of home measurements and because office measurements would be comparatively rare—but this design caused significant confusion as to why some points were disconnected. We decided on a final design connecting home and office BP measurements and relying on only two symbols to denote the source of the measurements (circles for home measurements and squares for office; Fig. 2C). While we decided to include office measurements in our display, we recognize that as home BP measurement becomes easier and more affordable, the relevance of office values may substantially decrease due to the greater robustness of home BP measurements and the relative infrequency of office measurements. The inclusion of office measurements in such a display may need to be reconsidered in future.

#### Goal ranges

**Fig. 3:**
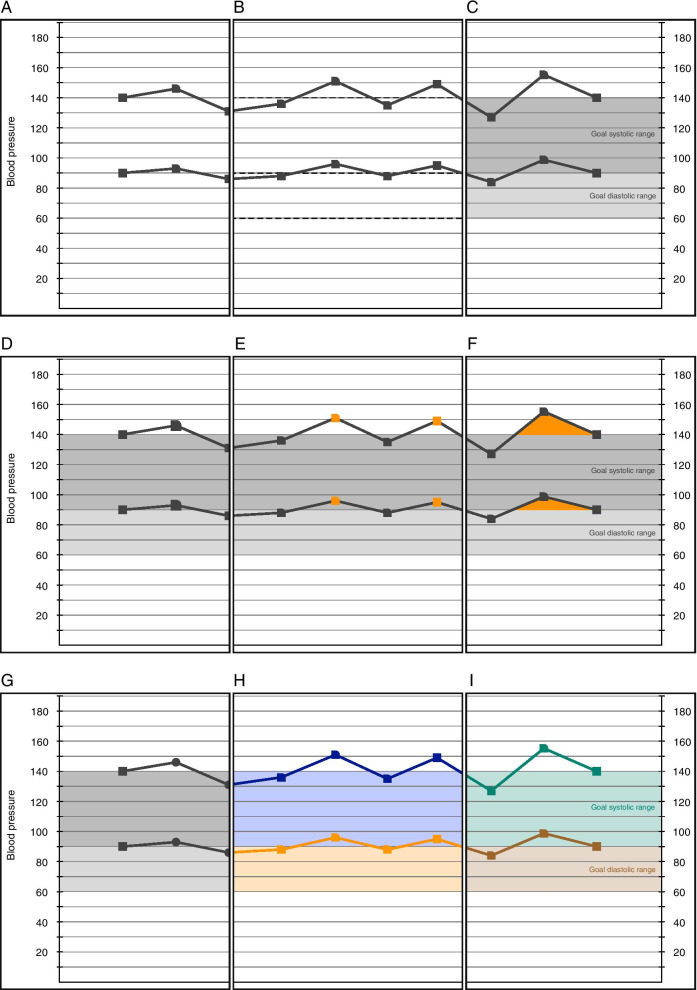
Examples of different approaches to illustrating BP goals ranges and how to communicate if a BP measurement is out of the goal range. **A** shows no goal range and **B** shows dotted lines as goal ranges; both approaches are used in EHRs and neither are informative for patients or physicians. **C** illustrates our greyscale goal range bands—two different shades of grey are used to denote the systolic and diastolic BP goal ranges, with no special affordances to denote out of range values. We tried several techniques to denote out of goal range BP measurements. **D** illustrates the use of larger symbols for out of range values; however, these were difficult to distinguish from in range values unless the symbols became obnoxiously large. **E** used colored symbols and **F** used colored fills below the line; however, neither option was well received by patients or physicians. **G**, **H** and **I** demonstrate our use of color to replace the grey bands and the introduction of the like-with-like paradigm—if the data points and connecting line are within the goal range band of matching color then the BP is in a healthy range; otherwise the BP is too high/low

The reader of the graph should be able to perceive at a glance whether the BP is in control or not. In existing EHRs, goal ranges may not be displayed at all (Fig. 3A) or may be shown only as horizontal lines (Fig. 3B). We added a band of background color to denote the acceptable ranges for SBP and DBP, making them visually more prominent than single horizontal lines (Fig. 3C). This design allows the visual cognition centers to employ the pre-attentive attributes of color and 2-dimensional position to quickly judge control [16].

Recommendations for goal ranges can vary for special adult populations (the elderly, those with diabetes, or chronic kidney disease) and may differ among guideline-issuing organizations, which also revise their guidelines every few years. Goal ranges may also need to be personalized for patients who have symptoms (dizziness from low BP), certain disease states (diabetes), organ dysfunction (renal impairment), or when BP varies so widely that it fluctuates beyond both upper and lower bounds. The numeric range covered by the goal range bands was pre-set the same for all patients at 90–140 SBP and 60–90 DBP. The focus group participants expressed a strong desire for the ability to personalize the BP goal ranges on a per patient basis. Later designs added those controls.

*“The shading allows us to quickly see if we’re below 140 and below 90 […] Do I get to adjust my shading for the risk of my patient?”*

**— Round 4, GIM Physician 7**

#### Out of range

A variety of methods were considered to provide visual emphasis to values out of range, including using larger (Fig. 3D) or colored points (Fig. 3E), or filling the area under the line—values above the upper normal range with the area under the curve filled a vivid orange color (Fig. 3F). Patients and physicians found those colors, weights, and fills distracting or unnecessary.

*“Right. It [orange squares] doesn’t seem to have a purpose. It doesn’t seem to clarify anything; it doesn’t seem to add anything to me.”*

*“… I can see that [orange fill means out of range]. I don’t need the orange.”*

**— Round 1, Patient 17**

#### Color

Initial designs used an all greyscale color palette (Fig. 3G). This allowed printing without loss of information and reserved color for adding visual emphasis only. Our goal range bands initially employed two different shades of gray for SBP and DBP, but users found them confusing. Focus group testing revealed a strong preference for color-coded goal ranges in patients.

*“There’s no color in the chart. It, the normal range of the graph is small and hard to read. It should be enlarged and blown up since those are the normal ranges.”*

**— Round 1, Patient 16**

We switched to pastel colors (more subtle, allowing use of more saturated colors for highlighting), with orange for SBP and blue for DBP, both colorblind safe (Fig. 3H). To avoid conflict with our EHR’s color standard for high (orange) and low (blue) values, a further iteration used a colorblind-safe two-color scheme—mint (#008471) was used for the systolic BP measurements and to denote the systolic goal range band; cocoa (#9C652B) was used for diastolic measurements and the diastolic goal range band (Fig. 3I). These latter two designs resulted in the creation of a *like-with-like* paradigm—if the data points and connecting line are within the goal range band of matching color then the BP is in a healthy range; otherwise the BP is too high/low.

*“Yes, the color is helpful. I think it’s gonna help, I think it would help patients to understand the difference between the diastolic range and the systolic range, and also you’re tying the blood pressure readings to the color of your target range. I think that is helpful.”*

**— Round 2, FCM Physician 10**

### Data and how to handle it

#### Data table

***“****I would probably almost want to put the numbers up there with the lines, you know, displaying, you know, what the numbers are…”*

**— Round 1, Patient 13**

Our original prototype designs did not include an associated data table as data tables are a universal component of EHR designs. However, at our focus groups, a proportion of both patients and physicians preferred to see the data table in addition to the graphical displays. For the data table, we adopted our host EHR color scheme and bold text (dual visual encoding) to denote out of range values. In the context of visually adjacent data tables and graphical displays, we initially assumed that for each data point, there should be a one-to-one correspondence between a table value and a graph data point. We encountered several considerations that caused us to question that assumption, namely issues with data density and missing data.

#### Data density

We believe we are currently in a transitional period for home BP measurements. In the past, we inhabited a period of *data dearth*—BP measurements were taken only during clinical visits in the office, or during hospitalization (whether hospital BPs are even available in an outpatient clinic setting is another story). Today we are in a period of *data nascence*—while a fraction of patients are able and willing to monitor their BP at home, their ability to upload those measurements to an EHR is not without technological barriers. We are rapidly approaching a period of *data abundance*—both wearable and non-wearable BP monitors become more ubiquitous and data standards evolve to allow for automatic and seamless uploading of regularly collected patient BPs directly in the EHRs of healthcare providers.

The potential for high density home BP measurements limits the numeric values that could be presented in the line graph and accompanying data table for any given length of time—very dense data will require aggregation. To address this, we designed a system of data aggregation to deal with dense measurements (Table 2). Users select how much time to show on the display (2 months of measurements, 2 years, etc.). The display should be able to comfortably accommodate up to 62 data points in the line graph and accompanying data table for any given length of time (see Table 2 for explanation). If a point represents > 1 measurement, there is no visual indication in the graph, but the value is presented in a bold typeface in the data table. While such a system will allow us to comfortably handle large amounts of data, it requires us to educate users that one point on the display does not necessarily represent one measurement.

**Table 2:** Data density

Time period to display	Maximum number of data points to display	Each point represents…	Comment
1 day	48	30 min	An Apple Watch measures the wearer’s heart rate every 10 min. It is not inconceivable of a future which includes wearables capable of passive BP monitoring at a similar interval. If we take a more extreme measurement rate of one measurement every 10 min, a single point on the display represents the average of three data points of recordings
1 week	56	3 h	Each day is represented by eight points on the display
2 weeks	56	6 h	Each day is represented by four points on the display
1 month	62	12 h	Each day is represented by two points, with a maximum of 31 days per month
2 months	62	1 day	The maximum number of days in any 2-month period is 62 (displaying July and August)
4 months	62	2 days	The maximum number of days in any 4-month period is 123
6 months	62	3 days	The maximum number of days in any 6-month period is 184
1 year	54	1 week	At most a year can have 366 days, so every point on the display could represent 6 days. However, thinking in terms of weeks is more common which would correspond to a maximum of 54 distinct weeks in a calendar year
2 years	54	2 weeks	–
3–5 years	60	1 month	–
> 5 years	60	2 months	–
> 10 years	60	4 months	–
> 15 years	60	6 months	–
> 30 years	60	1 year	–

#### Missing data

The potential for missing data is substantial currently, due to a combination of infrequency in office visits where measurements can be taken and the variable frequency of home measurements both within and between individual patients. We propose the following affordances to help account for missing data over a particular timeframe in the display: if < 10% of consecutive data points would be missing, we simply do not plot any points, connect the remaining points as we normally would, and leave gaps in the data table; if > 10% of consecutive data points are missing, we still leave gaps in the data table but we connect remaining points in the line graph with a dashed line. Thus, no points are plotted but the amount of missing data can be inferred from style of the line on the display (solid vs. dashed). This can be seen in Fig. 5—on the left, there is a large amount of missing data visible, denoted by a large gap in the data table and a dotted line in the graphical display; on the right, there is a small amount of missing data, denoted by a small gap in the data but a solid line in the graphical display.

#### Smoothing data

There is evidence that the variability of BP measurements has much less clinical significance than the mean BP [24]. Additionally, we previously found that patients place substantial weight on variability and outliers in BP measurements, despite the poor predictive value of those factors [18, 19]. To help address this, we used a locally weighted scatterplot smoothing (LOWESS) algorithm to smooth the data and added this smoothing line to the display (Fig. 5). The line graph of (raw) measurements was then faded slightly to help the smoothing line stand out. The properties of the LOWESS algorithm accounted for missing data as well. We conducted several experiments on patient perception of the smoothing line and found responses to be overwhelmingly positive [19].

### Annotations

We had discussed, and participants agreed, that recording behavioral changes (e.g., lifestyle changes) on the display, as well as metrics related to BP goals (e.g., weight, selected lab values), would be helpful.

*“Well, I’m trying to figure out how I would use it and the only thing I could see would be if there were some other intervention besides medication that occurred at that point. So you said this is where we started a restricted sodium diet or, you know, something like that.”*

**— Round 2, FCM Physician 18**

Participants were less receptive to the idea of the annotation presenting an analysis of the information that appeared obvious through the graphical representation (out of range, number of days BP was high/low, medication change) but analysis such as average BP over time was attractive.

*“…when you’re down in the grey area [goal range], which appears to be the range that you’re shooting for, well, you don’t really need the bubble to tell you you’re in there because you’re in there. So I just think, I don’t know that that adds anything to the body of the display.”*

**— Round 1, Patient 17**

**Fig. 4:**
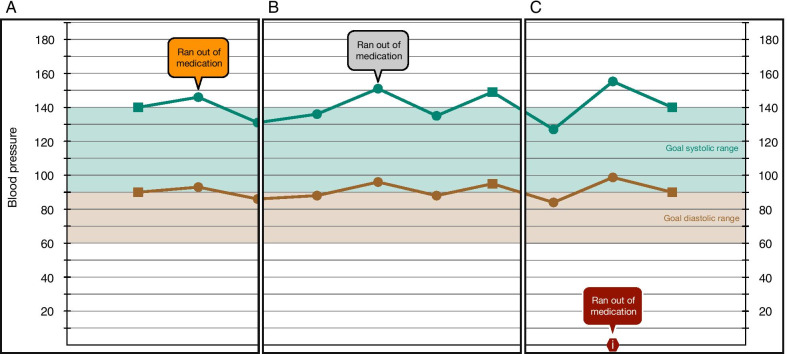
Examples of different ways user annotations could be displayed. **A** Our color-coded design, where the color of the annotation bubble was linked with the type of annotation content. This introduced too many new colors, so we used a single grey scheme shown in (**B**). Concerns over crowding of the display and the logic needed to decide the best location for the annotation bubbles led us to design the annotation timeline seen in (**C**). Here, the presence of annotations is denoted on the x-axis of the graphical display and the annotation bubble appears only when a user hovers their cursor over it

We experimented with two approaches to display user-generated annotations. First, we explored a free-floating-bubble design where annotations were displayed as floating bubbles that would hover over the various parts of the display. Two schemes were used: (1) a color-coded variant, in which different colors of annotation bubbles denoted different topics (Fig. 4A); and (2) a grey variant, in which all bubbles were grey (Fig. 4B). It was eventually determined that the grey variant was preferable—the colored version introduced too many colors to the display.

However, a potential concern of the free-floating-bubble design was if annotations became popular, they would overwhelm the screen. Additionally, this design introduced certain problems: Where do you put the bubble? Does the bubble have to be manually placed by the person making the annotation or is there some internal logic that is used?

To avoid these issues, an annotation timeline was introduced (Fig. 4C). Annotations were denoted by a symbol placed on this x-axis of the graphical display. When a user hovers over the symbol, the text of the annotation appears. This second design had several advantages: (1) reusing the x-axis of the graphical display meant we did not have to increase the complexity of the display; (2) hiding annotations until they are hovered over minimized visual clutter; and (3) the design was kept simple—a user only had to enter a date and the content of the annotation.

### Medication timeline

**Fig. 5:**
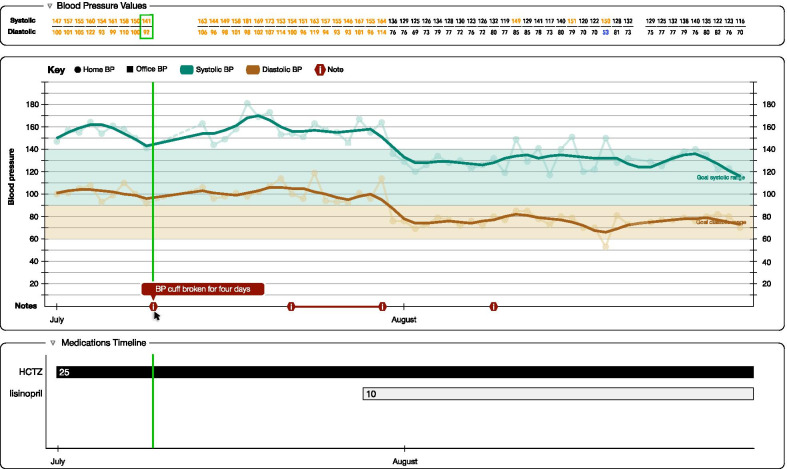
Mock-up of our entire display, including the graphical display and smoothing line, annotations, data table, medication timeline, and scrubber bar

We previously designed a medication timeline to help visualize polypharmacy, which was found to improve physician performance in routine medication management (e.g., finding the start date of a new medication), compared to more traditional tabular displays of medication lists [25]. We incorporated this medication timeline into our designs for this display (Fig. 5). Many patient focus group participants demonstrated an intuitive understanding of the display and were able to derive meaning across the main BP line graph and the supplemental tables. This linking of data allowed them to draw conclusions about how changes in medications seems to correlate with changes in BP.

*“I mean, yeah, it looks like when they started the Lisinopril that, you know, the blood pressure started going down and stuff from the 10, low dose of a 10 to a 20, so they had to increase it to keep it down.”*

**— Round 3, Patient 1**

### Scrubber bar

In many modern EHRs cross-categorical displays—which show all these elements in a single display—are rare and there is a need to switch between displays, which increases cognitive load. Our display presents the graphical display, data table, and medication timeline in a single display. We introduced a colored, vertical scrubber bar to help both patient and physician users link these elements together (Fig. 5). As a user moves their mouse, the vertical scrubber bar tracked the horizontal mouse movements of the user’s cursor across all parts of the display (i.e., graphical display, data table, and medication timeline). Users indicated the scrubber bar allowed them to more easily link the data ‘story’ across the graphical display, data table, and medication timeline.

*“I like it, because when you move the mouse it then shows you at this point, this is your blood pressure, this is the medicine you were on or a combination of medications.”*

**— Round 1, Patient 17**

## Discussion

Our goal was to design a graphical display to visualize temporal trends for an individual patient's blood pressure, to support shared decision making between the patient and their healthcare provider with the aim of improving BP control across the population. The primary audience for this display is the patient-provider dyad (or caregiver-provider dyad) whose shared goal is to achieve the long-term goal of improved hypertension control. The display needed to clearly show whether BP was controlled compared to a goal range, to make recent trends in the BP apparent, to support judgments to improve control, and to foster action to achieve control. Design decisions were made with the intent of minimizing cognitive load by using principles that leverage fast visual cognition [16]. Table 3 presents a summary of all design features, related design decisions, and rationale for each decision. The focus group responses from patients and physicians confirmed the effectiveness of those choices. The design was perceived as generally intuitive and brief orientation to the tool tended to clarify any remaining ambiguities.

**Table 3:** Design features, decisions, and rationale

Design feature	Final design decision	Rationale [Source]*
*Blood pressure plot*		
Style of plot	Use a line graph	Unnecessary to introduce a novel design. Use a format with which all users are already familiar. [RT]
Denoting specific sources of BP readings	Use two symbols to denote BP data as “home” or “office” readings	Unique symbols for additional possible data sources—beyond home and office measurements—could lead to confusion. [RT] Physicians reported specific differentiation beyond home and office measurements was unnecessary as some sources are already evident due to their density (e.g., high density inpatient or operative values). [FG]
	2-line paradigm (systolic and diastolic), mixing both home and office readings, with the source differentiated by two symbols	4-line paradigm could result in overlap and visual confusion. [RT]
Goal ranges	Add filled goal range bands on the graphical display to denote systolic and diastolic goal ranges	Allows of the visual centres of the brain to employ the pre-attentive attributes of color and 2-dimentsional position to judge control at a glance [16]. [RT]
	Create goal ranges which are adjustable on a per patient basis	Goal ranges can vary for special populations or for patients with specific symptoms. [FG]
Out of range	No additional affordances aside from data points being outside the goal range bands	Additional affordances, such as colored points or fills, were found to be distracting or unnecessary. [FG]
Color	Add colored goal range bands which match colored systolic and diastolic points and lines	Strong patient preference for use of color. [FG] Creates a like-with-like paradigm for fast visual processing to determine if points are within or outside goal ranges [16]. [RT]
	Use a two-color scheme of mint (#008471) for systolic BP measurements and cocoa (#9C652B) for diastolic measurements	Color scheme needs to be colorblind-safe and avoid conflict with existing color scheme in the EHR. [RT]
*Data and how to handle it*		
Data table	Include a data table to show the corresponding values for each point on the graphical display	Preferences of both patients and physicians for the inclusion of a data table with the measurement values. [FG]
Data density	Use a display which can accommodate 62 data points	A future with high density home BP measurements will require a display of sufficient size to aggregate and visualize the data. A proposal for data density was developed and presented in Table 2. [RT]
Missing data	Use a dashed line on the line graph when > 10% of consecutive data points are missing	The current potential for large amounts of missing data is high as home BP measurement is only beginning to become more common and missing data needs to be clearly denoted. [RT]
Smoothing data	Use a LOWESS algorithm to smooth data and add smoothing line to graphical display	Patients overweigh the impact of variability and outliers in their BP measurements [18, 19] while variability has been shown to have much less clinical significance, compared to mean BP [24]. [RT]
Annotations	Add an annotation timeline onto which use-generated annotations can be organized	The research team, patients, and physicians recognized the value of user-generated annotations for tracking behavioral changes impacting BP not easily captured elsewhere. [RT, FG]
Medication timeline	Incorporate a medication timeline so users can understand the impact of medication changes on BP measurements	The medication timeline we previously designed [25] can provide users with additional context which is currently unavailable. Patients found the timeline was intuitive to use and provided additional context to their BP measurements. [RT, FG]
﻿Scrubber bar	Use a scrubber bar which links the various elements of the display	Patients found the scrubber bar helped link the various elements of the display (graphical display, data table, and medication timeline) into a more coherent story. [RT, FG]

*FG, Focus group; HC, Human factors and cognition; RT, Research team

We partnered with the Tiger Institute, a technology collaborative between the University of Missouri and Cerner Corporation, to produce and deploy this as a separate display view in our local EHR implementation using the SMART on FIHR platform. The SMART (Substitutable Medical Apps and Reusable Technology) project (https://smarthealthit.org) harnesses the FHIR (Fast Healthcare Interoperability Resources) from HL7 (Health Level 7) to create open solutions and tools for innovators to build applications that can connect to systems using the FHIR platform. Using this platform or FHIR standards, non-commercial or commercial developers could deploy the design into other EHRs or Personal Health Record platforms.

We explored several considerations where data was out of the default goal range or missing. Our guiding principles were data transparency and visual simplicity. For making effective treatment decisions, adding complexity or granularity seldom improved patient or provider understanding of degree of control or whether action was needed. The users valued intuitiveness over fine detail.

Smoothing the data in the line graph allowed us to emphasize mean blood pressure trends—which most accurately predict clinical outcomes—making degree of BP control more apparent to our users. Not only is the mean more apparent but so are changes in the BP trend. The smoothing line helped patients and physicians deemphasize variability fluctuations that had low clinical significance.

### Limitations

First, the work was done in a single academic health care organization in two primary care departments (family medicine and general internal medicine) where the working prototype is deployed in a single EHR. However, this tool has promise for scaling across the non-commercial or commercial EHR environment as the designs could be integrated given the right technical supports. Second, we took a primary care perspective during the design process as the majority of hypertension care is provided in the primary care setting [26]. However, we recognize that other specialist perspectives may have different requirements for how best to visualize BP measurements.

### Future directions

Further efforts should include adding the ability to modify the goal range for special patient categories (diabetes or chronic kidney disease), for different age groups (pediatric and geriatric), and to individualize for patients who have symptoms (dizziness from low BP) or other special characteristics. We recommend further work to include provider or patient annotations within the timeline, and to develop machine language linguistic summary annotations that would foster action to improve BP control (e.g., “Contact your provider soon to consider treatment changes to improve BP control.”).

## Conclusions

We iteratively developed a data display specifically designed to encourage shared decision making between hypertensive patients and their healthcare providers. This could help us overcome the clinical inertia that often results in a lack of treatment intensification, thus leading to better care for the 35 million Americans who have hypertension but do not have their blood pressure under control.

## Data Availability

The data that support the findings of this study are available from the corresponding author, PW, upon reasonable request.

## Abbreviations

BP: Blood pressure
DBP: Diastolic blood Pressure
FCM: Family and community medicine
EHR: Electronic health record
FHIR: Fast Healthcare Interoperability Resources
GIM: General internal medicine
HL7: Health level 7
JNC-8: Joint National Commission on Hypertension
LOWESS: Locally weighted scatterplot smoothing
SBP: Systolic blood pressure
SMART: Substitutable medical apps and reusable technology
USPSTF: United States Preventive Services Task Force
